# Comparative Analysis of the Comfort of Children and Adolescents in Digital and Conventional Full-Arch Impression Methods: A Crossover Randomized Trial

**DOI:** 10.3390/children11020190

**Published:** 2024-02-02

**Authors:** Diego Serrano-Velasco, Andrea Martín-Vacas, Patricia Cintora-López, Marta Macarena Paz-Cortés, Juan Manuel Aragoneses

**Affiliations:** 1 PhD Program in Translational Medicine, Universidad San Pablo—CEU, CEU Universities, 28003 Madrid, Spain; polackdiego.serranovelasco@usp.ceu.es; 2 Faculty of Dentistry, Alfonso X El Sabio University, 28691 Madrid, Spain; amartvac@uax.es (A.M.-V.); pcintlop@uax.es (P.C.-L.); jaraglam@uax.es (J.M.A.); 3 Department of Dental Research, Federico Henriquez y Carvajal University, Santo Domingo 10106, Dominican Republic

**Keywords:** pediatric dentistry, orthodontics, patient comfort, dental impression technique

## Abstract

The aim of this study was to evaluate the comfort of children and adolescents with conventional full-arch dental impression methods compared to two intraoral scanners (iTero^TM^ and Primescan^TM^). Methods: A monocentric, analytical, controlled crossover study was designed to compare conventional impression and digital impression with two intraoral scanners (iTero^TM^ and Primescan^TM^) in children and teenagers. Patient comfort was evaluated using a 100 mm VAS scale adapted to Spanish and for children. A descriptive and analytical statistical method was conducted with a confidence level of 95% (*p* ≤ 0.05) and asymptotic or bilateral significance. Results: A total of 51 subjects were enrolled in the study (mean age = 12.35 years). Although the group of 10–14-year-olds was the most numerous, gender was equally distributed among the age groups. None of the variables on the VAS scale showed differences between the gender categories (*p* > 0.05). There were differences (*p* < 0.05) with respect to the age categories, as the middle adolescent group showed the worst general perception and total comfort during the conventional impression. Statistically significant differences were found between all VAS scale items and the three impression methods (*p* < 0.05). Conclusions: The digital impression technique is superior in terms of total comfort to the conventional alginate impression in children and adolescents.

## 1. Introduction

The reproduction of oral structures with an impression and its subsequent printing on a cast is one of the most frequent procedures in the dental office, since it is necessary for the diagnosis, treatment plan, and preparation of oral prostheses and orthodontic appliances. Intraoral scanners were introduced in dentistry in the 1970s of the 21st century [1], and in 1999, the first orthodontic scanner system was created (OrthoCAD version 1, Cadent Technologies Inc., Carlstadt, NJ, USA). In recent times, multiple intraoral scanners have appeared with the aim of digitizing the dental impression process. Currently, the existence of dental scanning methods is presented as an alternative to conventional dental impression methods, with benefits such as greater comfort for the patient and adequate reliability [2,3,4,5,6]. However, the costs are high, and a greater learning curve is necessary [7].

The most frequent disadvantages of conventional impression are discomfort during the process, nausea, temporomandibular joint (TMJ) discomfort, time perception, and shortness of breath, among others [8,9,10,11]. In a systematic review [12], the authors confirm that all articles prove benefits in favor of intraoral scanning over the conventional technique, despite showing no changes in the level of perceived anxiety between both methods. In general, the positive sensations perceived with intraoral scanners are related to smell, taste, sound, vibration, nausea, and queasiness, although the evidence is of variable quality. A recent umbrella review evaluated conventional and digital implant impressions and concluded that nearly all reviews agree that patients prefer using digital technology over traditional impression methods, and intraoral scanning appears to be more efficient in relation to the time needed to take the impression. However, as the authors state, the results obtained are influenced by numerous factors [13].

The child and adolescent populations are groups with a high demand for orthodontic treatments. Furthermore, they stand for a group that is more susceptible to discomfort or fear in dental consultations. Some authors have already conducted studies on pediatric patients, obtaining a better understanding of the digital method compared to the conventional one [8,10,14]. Regarding anxiety during both procedures, intraoral scans offer positive results in terms of feelings of uneasiness, insecurity, fear, nervousness, and happiness [9].

Nowadays, taking impressions continues to be a procedure that generates rejection and discomfort for patients in general, with pediatric patients being the most susceptible to experiencing discomfort or fear in the dental office. That is why the idea of being able to replace conventional prints with digital ones is interesting. Since children have different sensitivity than adults and the reproduction of smaller oral structures with the same devices can be complicated, a study is necessary that analyzes children’s perception of comfort during dental scanning procedures. Due to the theoretical framework, we proposed the null hypothesis that the comfort perceived by children and adolescents does not differ between the conventional and digital impression methods with intraoral scanners (iTero^TM^ and Primescan^TM^) nor between the two intraoral scanners studied. The aim is to evaluate the comfort of children and adolescents with conventional full-arch dental impression methods compared to two intraoral scanners (iTero^TM^ and Primescan^TM^).

## 2. Materials and Methods

### 2.1. Trial Design

This is a monocentric, controlled, randomized crossover trial. The experimental design followed the Consolidated Standards of Reporting Trials (CONSORT) statement and extension checklist for crossover randomized trials [15].

### 2.2. Ethics Statement

The present study was accepted by the Ethics Committee of the San Carlos Clinical Hospital (code 21/336-E) and complies with the requirements of the Declaration of Helsinki for biomedical research.

### 2.3. Participants and Sample Size

The subjects were enrolled in the study at the Master’s Degree in Orthodontics clinic at the Alfonso X El Sabio University, Spain. The sample size was calculated using G*Power 3.1.9.7. with the procedure for comparing repeated measures within the factors, with a single group and three measurements. A total of 43 subjects would be needed to determine a medium effect size (0.25) with an alpha error (*p*) of 0.05 and 95% power. To avoid a possible loss of 25% of the subjects, it was decided to increase the initial sample to 60 subjects.

Through non-probabilistic sampling of consecutive cases, 62 patients who attended the Master’s Degree in Orthodontics clinic at the Alfonso X El Sabio University (UAX) were invited to participate, meeting the established inclusion and exclusion criteria. To be included in the study, they had to be (1) less than 18 years old, (2) have no experience in dental impression techniques or intraoral scanning, and (3) request an occlusion study in the Master’s Degree in Orthodontics clinic at the UAX. We excluded (1) subjects with craniofacial syndromes, (2) systemic diseases, (3) behavioral problems, or (4) psychological disorders presenting with intellectual disability.

Written informed consent was given to the parents and/or legal guardians of the children, obtaining acceptance from the child when he or she was over 12 years old, in accordance with current regulations on the protection of personal data (Constitutional Law 3/2018 and General Data Protection Regulation -EU- 2016/679 of the European Parliament and of the Council).

The final sample was formed of 51 patients, since 11 finally refused to participate in the study or could not be contacted.

### 2.4. Interventions

The orthodontic study in the Master’s Degree in Orthodontics clinic at the UAX is protocolized, being composed of a conventional full-arch alginate impression (for the preparation of plaster study models) (Orthoprint—Zhemarck SpA, Badia Polesine, BO, Italy) and an intraoral scan with iTero Element^TM^ 5D Plus (^© 2024^ Align Technology, Inc., San José, CA, USA). At that appointment, the existence of this study was explained to the parents or legal guardians, giving them a patient information sheet and explaining that they would be called to ask if they wanted to take part in the study. All patients who were part of the eligible population were assigned successive numbers with the delivery of the study information sheet. To avoid bias due to the order of the procedure, it was decided through simple randomization whether the conventional impression or the intraoral scan with iTero^TM^ was performed first.

**Figure 1:**
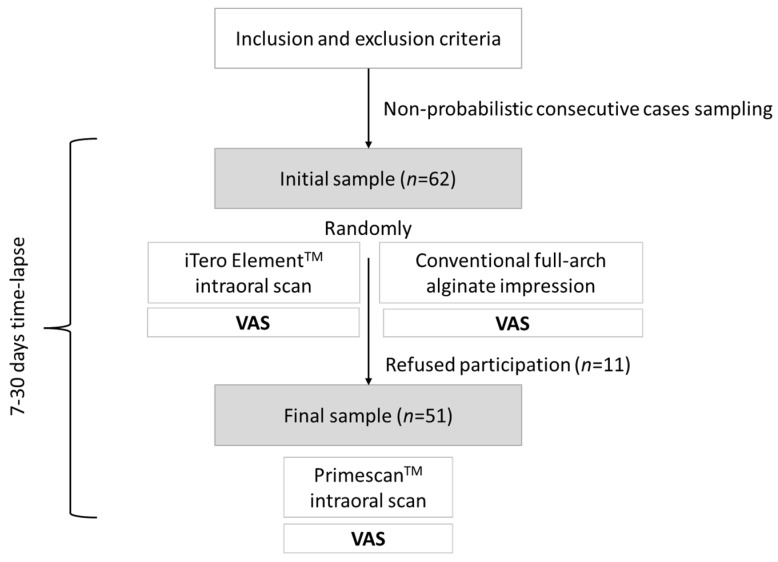
Study sample selection and distribution of the interventions.

Parents and/or guardians of patients who were part of the eligible population were contacted to ask if they agreed to be part of the study, and if so, they were scheduled a second time to be scanned with the Primescan^TM^ intraoral scanner (PrimeScan^TM^, Dentsply-Sirona^TM^, New York, NY, USA). The second appointment was separated between 7 and 30 days from the first appointment to avoid interference in the assessment of the comfort of the children and adolescents. At the second appointment, informed consent was given, and they were scanned with Primescan^TM^. Those patients who refused to take part in the study or could not be contacted were eliminated from the data analysis, and the first VAS registers were deleted. The process of selecting the study sample and the distribution of the interventions can be seen in Figure 1.

The impressions (both digital and conventional) of the first day were taken by the students of the Master’s Degree in Orthodontics at the UAX, always supervised by a professor, as they were part of the procedure of the orthodontic study. The impression of the second day was always taken by the principal investigator of the study (D.S.-V.). Both the students of the Master’s Degree in Orthodontics and D.S.-V. were trained to take impressions, both digital and conventional, during the early stages of their training, and, therefore, measurement biases were avoided.

Conventional alginate impressions of both arches were taken with steel impression trays, and red wax was used for bite registration. The alginate was hand-mixed with tap drinking water. The procedure was as follows: (1) test of the tray size; (2) preparation of the alginate according to the manufacturer’s instructions; (3) impression of the lower arch; (3) preparation of the alginate according to the manufacturer’s instructions; (4) impression of the upper arch; and (5) bite registration. Intraoral scans were performed following the guidelines of each manufacturer with the patient lying down following the same sequence: lower arch, upper arch, and bite registration, according to each manufacturer’s instructions.

### 2.5. Randomization

On the first day, the order of the two main procedures (digital or conventional full-arch impression) was simply randomized, so that 29 subjects received the conventional impression method as the first impression and 32 received the digital impression with iTero^TM^ as the first impression. The random method, since the sampling was of consecutive cases, was conducted simply using a coin. The second appointment always included a digital intraoral scanner with Primescan^TM^, which is not part of the occlusal study.

### 2.6. Outcomes

The WHO defines the adolescent period as the period between childhood and adulthood, from ages 10 to 19. Neistein [16] and Brañas [17] in the 1990s divided adolescence into three stages: first adolescence (10–14 years), middle adolescence (15–17 years), and late adolescence (18–21 years). In our case, we classified the study groups into three categories depending on age measured in years: children (<10 years), early adolescence (10–14 years), and middle adolescence (15–17). The children were also classified according to their gender as female, male, and others.

The primary outcome of the trial was patient comfort. The comfort of the subjects was evaluated immediately after the impression/scanning with a VAS scale, adapted to Spanish by Yilmaz et al. [10], in which the child was instructed to mark with a cross how he or she felt during the procedure regarding general perception, breathing, smell–taste, heat–cold, nausea, gag reflex, and pain. The VAS records of the patients who refused to continue with the study or who did not come/answer the phone for the second scan were removed. Due to the different maturity levels of the age groups, the VAS scale was filled out by the child, supervised by the operator, using guided questions in case they did not understand the filling method. The degrees of these parameters were recorded using a 100 mm VAS index, which was supported with 11 facial emojis designed specifically for children instead of the classic visual analog scale (VAS) [18]. The scale includes a smiling face as the lowest score (0 mm) and a crying face with tears as the highest score (100 mm), with nine equidistant intermediate faces. The VAS scale measurements were always carried out by the same observer (D.S.-V.) and the same electronic digital caliper (Vietnam E-Commerce Limited, RM 18 27/F, Ho King, COMM CTR 2 16 FA Yueng St. Mongkok, Kowloon, Hong Kong) (Technical Specifications in Table 1). To avoid eye strain, a maximum of five pairs of models were measured per session, always in the same room with the same artificial light and closed blinds, avoiding interference from natural light.

### 2.7. Blinding

Due to the distinctive characteristics of conventional and digital impressions, both the operator and the patient could not be blinded during the technique. To keep the study anonymous, the initial results of the VAS scale were hidden from the operator at the patient’s second visit. The data were tabulated by another researcher, blinded to the procedures (A.M.-V.), to avoid bias.

### 2.8. Statistical Methods

The calculation of the sample size was performed with G*Power, as previously established. An analysis of the intraobserver agreement of the VAS measurements on children’s comfort was carried out in a random 10% of the sample collected with the intraclass correlation coefficient (ICC) with a two-factor mixed model, absolute agreement procedure, interpreted according to Koo and Li [19].

Descriptive statistics were performed to describe the quantitative variables (mean and standard deviation) and the qualitative variables (frequency and percentage). The distribution of age in the study sample was analyzed with a Student’s *t*-test for one sample, and the distribution of gender and age group variables was analyzed with a chi square (ꭓ^2^) test. The adjustment to a normal distribution was evaluated with the Kolmogorov–Smirnov (with the significance correction of Lilliefors) and Shapiro–Wilk tests. Because the comfort evaluation did not follow a normal distribution in any of the measured variables, non-parametric tests were carried out to contrast hypotheses. We evaluated whether age or gender affected the comfort of the type of impression between the conventional impression and intraoral scanner with the Mann–Whitney U test (gender) and Kruskal–Wallis test (age group) for independent samples. To contrast the hypotheses of the evaluation of comfort in the three impression/scanning methods used, the Friedman two-way analysis of variance test for related samples was used. The significance values of post-hoc tests for a pairwise comparison of the variables were adjusted with the Bonferroni correction in several tests.

The tests were carried out with the SPSS 24^®^ Software (version 24.0, Armonk, NY, USA) with a confidence level of 95% (*p* ≤ 0.05) and asymptotic or bilateral significance.

## 3. Results

**Figure 2:**
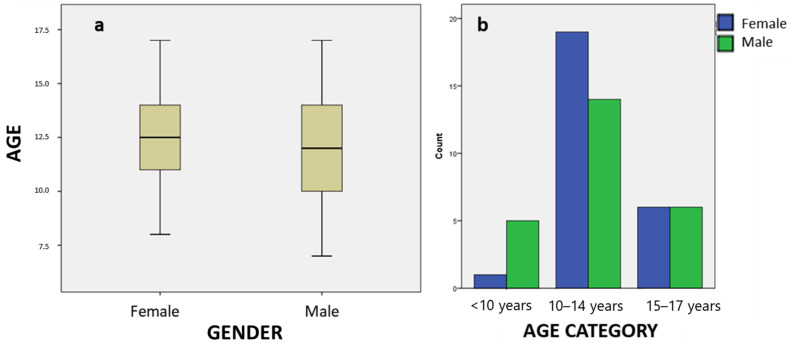
Boxplot of age and gender of the study subjects (**a**) and bar chart of distribution of the age category groups (in years) of the study sample classified by gender (**b**).

A total of 62 children were invited to participate in the research; however, 11 refused participation. A total of 51 subjects were studied, with a mean age of 12.35 years (SD = 2.57, minimum = 7, maximum = 17). Age followed a normal distribution (Kolmogorov–Smirnov *p* = 0.200, Shapiro–Wilk *p* = 0.237). When classifying the subjects into age groups, an asymmetric distribution was seen (chi square *p* < 0.001), with children between 10 and 14 years old predominating (*n* = 33) over those under 10 (*n* = 6) or over 14 (*n* = 12). Regarding the distribution by gender, it was balanced (girls *n* = 26, boys *n* = 25). It was analyzed whether age differed in the gender distribution of the sample, finding that there were no statistically significant differences between the mean ages of girls (mean = 12.77, SD = 2.18) and boys (mean = 11.92, SD = 2.9). (t-test *p* = 0.242). The distribution by gender within the age groups was also homogeneous (Fisher *p* = 0.218) (Figure 2). The intraoperator agreement in the measurement of the VAS scales was analyzed, finding excellent reliability in all cases.

The seven components of the VAS scale were described to analyze the comfort of the study subjects, and their average was calculated to obtain the general comfort for each dental impression medium. The values can be seen in Table 2. None of the variables showed a normal distribution (Kolmogorow–Smirnov and Shapiro–Wilk *p* > 0.05 in all cases).

**Figure 3:**
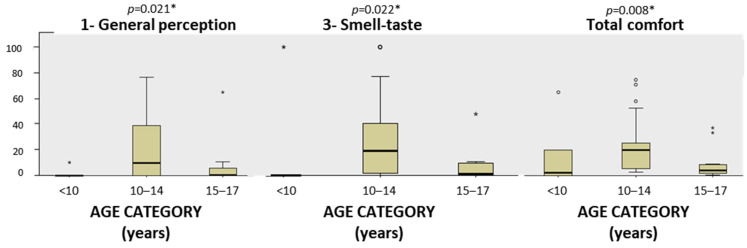
Box diagram of Item 1 (general perception), Item 3 (smell–taste), and total comfort of the VAS scale of conventional impression. * Kruskal–Wallis test for independent samples. Statistically significant, *p* ≤ 0.05. Circles and asterisks represent atypical and extreme values, respectively.

It was analyzed whether the gender and age categories influenced the comfort reported by the study subjects. The results showed that none of the variables of the VAS scale analyzed showed significant differences in the distribution between the gender categories (Mann–Whitney U *p* > 0.05 for all cases). On the other hand, it was found that with respect to the age category, there were statistically significant differences exclusively in the conventional impression for Item 1 (general perception) (Kruskal–Wallis *p* = 0.021), Item 3 (smell–taste) (Kruskal–Wallis *p* = 0.022), and total comfort (Kruskal–Wallis *p* = 0.008). Post-hoc comparisons were made between pairs on the significant variables, finding that the differences in Item 1 (general perception) were significant between age categories 1 (children) and 2 (early adolescence) (adjusted *p* = 0.050), while for total comfort, the results were significant for age categories 2 (early adolescents) and 3 (middle adolescents). In both cases, it was significant that the middle adolescent age group showed greater discomfort than the other groups for Item 1 (general perception) and total comfort in the conventional impression. For Item 3 (smell–taste), post-hoc tests with adjusted significance ruled out significant differences (adjusted *p* = 0.095) (Figure 3).

**Figure 4:**
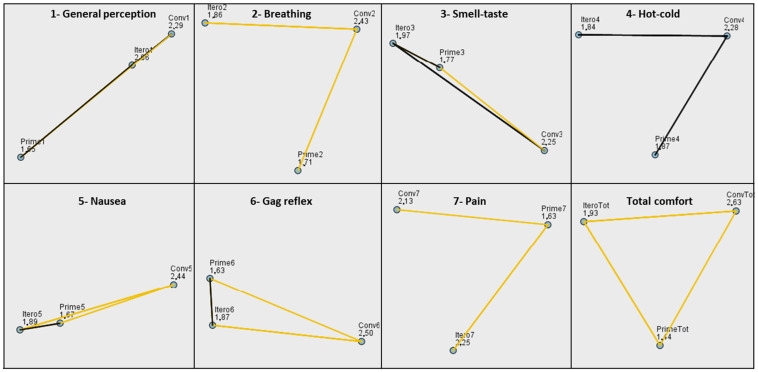
Post-hoc comparisons between pairs of variables for each item of the VAS scale in the impression methods analyzed. Each node shows the average range of samples. The yellow lines stand for statistically significant results of the post-hoc paired test (*p* ≤ 0.05).

Comparisons were made for related samples to analyze all components of the VAS comfort scale. Statistically significant differences were found between all VAS scale items (Friedman *p* < 0.05 for all comparisons). Paired-sample post-hoc tests were performed to analyze the results (Table 3, Figure 4). Greater discomfort was found for general perception (Item 1 VAS), smell–taste perception (Item 3 VAS), and pain perception (Item 7 VAS) with conventional impression than with Primescan^TM^ but not with iTero^TM^. Items 2 (breathing), 5 (nausea), and 6 (gag reflex) showed greater discomfort from the conventional impression compared to the two intraoral scanners, iTero^TM^ and Primescan^TM^. Item 4 (hot–cold) did not show differences between any of the impression methods in the post-hoc tests with adjusted significance. The only variable of the VAS scale in which the two intraoral scanners differed from each other is for Item 7 (pain), since the study subjects perceived the scan with iTero^TM^ as significantly more painful than with Primescan^TM^. When analyzing the total comfort obtained from the average of the scores obtained on the VAS scale, significant pairwise differences were found between the three methods, such that the most comfortable method was the Primescan^TM^ intraoral scan, followed by iTero^TM^, and, finally, conventional impression.

## 4. Discussion

In recent years, the use of digital intraoral scanners has increased significantly in dental clinics. The aim of the study was to evaluate the comfort of children and adolescents with intraoral scanning procedures compared to the conventional method by comparing a widely used and studied scanner (iTero^TM^) with another recently commercialized one (Primescan^TM^). As reported in recent systematic reviews, intraoral scans seem to be a promising method for both adult [12] and pediatric [20] patients. Although children and adolescents make up most patients susceptible to orthodontic treatment, many studies conducted on perception, comfort, and preference have been performed on adult patients [3,4]. In the present study, 51 subjects are studied in terms of comfort using a VAS scale, obtaining favorable results for the digital method compared to the conventional one.

Before beginning the discussion of the data, it is relevant to analyze the method carried out in comparison with other authors who have studied children and/or adolescents. Regarding the intraoral scanners studied, we considered that iTero^TM^ should be one of them, since it is perhaps the most used one internationally. Primescan^TM^ is a new intraoral scanner that offers certain advantages, such as its speed and small head, and, therefore, we found it interesting to study. Yilmaz and Aydin [10] studied the 3-Cart Color Trios^®^ (3Shape) compared to conventional printing in 30 children. Glisic et al. [9] compared Trios Classic (3Shape, Copenhagen, Denmark) in 59 children and teenagers. In a recent study, 24 children scanned with Trios^®^ 3 (3Shape) were evaluated in comparison with conventional printing [11]. A study similar to ours, in which two scanners (Lava COS and Cerec Omnicam) are analyzed, is the one carried out by Burhardt et al. with 38 children [8].

Regarding the comfort analysis method, the one used in this study (100 mm VAS scale) was translated from the one used in the study of Yilmaz y Aydin in 2019 [10]. Similar to our study design, Glisic et al. [9] used a 100 mm VAS to evaluate comfort (seven questions) and anxiety (six questions). In Bosoni et al.’s study [11], they analyzed comfort with a VAS scale with the Wong–Baker scale to evaluate the duration of the procedure, comfort, pain, gag reflex, and respiratory difficulty. In another study, they analyzed comfort using a Likert-style perception questionnaire with five points to evaluate gag reflex, breathing, sensation of comfort, duration of the procedure, and stress during the procedure [8]. Concerning the age of the study sample, the mean age in the study was 12.35, similar to that of Burhardt et al.’s study [8] (mean = 12, range 10–17) and Glisic et al. [9] (girls’ mean = 12.83 and boys’ mean = 12.56), but higher than that studied by Bosoni et al. [11] (mean = 8.8, SD = 1) and Yilmaz and Aydin [10] (mean = 10.16, SD = 1.77).

According to the results found in this research, overall patient comfort is significantly higher with the intraoral scanner than with the conventional impression [8,9,10,11]. Specifically, according to our results, the best method in terms of comfort was Primescan^TM^, followed by iTero^TM^, and, finally, the conventional alginate method.

Yilmaz and Aydin evaluated 3-Cart Color Trios (3Shape) [10], finding better comfort with the digital method in the scores of general discomfort, smell–taste, gagging or gag reflex, and total score; however, there were no differences in the variables of respiratory distress or pain. Consistent with Yilmaz’s results, Bosoni et al. [11] found significant differences in gag reflex and respiratory distress but found no differences in pain. On the other hand, Glisic et al. [9] state that significant differences are found in all the studied outcomes (gag reflex, breathing difficulty, smell/sound, and taste/vibrations), except temperature and time perception. Our results differ in some outcomes. We found the best experience with Primescan^TM^ compared to the conventional method in terms of general perception, smell–taste perception, and pain perception, with no statistically significant differences with iTero^TM^. Perception of breathing, nausea, and the gag reflex showed the same improved experience with both intraoral scans compared to the alginate impression. There was only one outcome that differed between both intraoral scans—pain—because the subjects reported more pain perception during the iTero^TM^ than during the Primescan^TM^ scan.

Furthermore, about the comfort perceived by the operator, a better score for comfort was also obtained with the digital method than the conventional one [10], both in general comfort and in movement of the child, difficulty breathing, vomiting, clenching, or retching, with no differences in crying between both methods. Burhardt et al.’s results [8] are interesting since the children had more nausea in the upper impression with alginate than with the CEREC Omnicam, with no differences in the rest of the variables analyzed either between the two intraoral scanners or with the impression with alginate.

Although it is not the subject of the study, in terms of comfort, there are studies in adult patients also finding benefits in terms of comfort in the use of intraoral scanners. In Yuzbasioglu et al.’s study in adult patients [4], they compared the levels of stress and anxiety in a conventional impression with polyether and digital impressions (CEREC Omnicam, Sirona), obtaining significant favorable results in terms of comfort and preference in the digital impression group. Burzynsky et al. conducted a study with subjects 8–56 years of age (mean age = 15 years) without differentiating between children and adults [3]. In a study similar to ours, they evaluated two digital intraoral scanners (iTero^TM^ Element and TRIOS Color Intraoral Scan) with the conventional alginate impression. In their study, they found that patients felt less pain with iTero^TM^ than with the other printing methods. In a study comparing polyvinyl siloxane vs. a scanner (3M True Definition), perception was better with the digital technique than with the conventional one in terms of taste/smell, reflex gag, and general preference [21].

It seems clear, therefore, that the preference between both methods favors the digital method over the conventional one. According to the study carried out by Bosoni et al. [11], 75% of children prefer intraoral scanning. In Burhardt et al.’s study [8], the percentage of preference for the digital method drops to 51%, while 29% prefer conventional printing and 20% have no preference. This trend seems to not be maintained in studies carried out in adults, since in a study, 73.3% of patients preferred the conventional impression with alginate over the digital one (LAVA COS) because it was easier or faster, while 26.7% preferred the intraoral scans for being more comfortable [22]. In another study carried out with Carestream CS 3600 [23], 91% also preferred conventional printing, without differences in age, gender, or order of interventions. Furthermore, following the line of the study, they found a relationship between the preferred procedure and the one they had considered most comfortable.

The present study has some weaknesses. Firstly, the collaboration of the youngest children in completing the VAS scale was less, so in those cases, the professional had to help them more to complete the scale, and there may be biases in the child–professional communication. Furthermore, the use of VAS scales always entails a small margin of error, since it depends on the sensitivity threshold of each patient to the stimuli evaluated. Another possible bias is that there is no group in which the two scans were performed first and then the impression, since we consider that performing both scans at the same appointment would be confusing for evaluating comfort. In addition, there is an important limitation in the study related to the impossibility of blindness. The operators were evidently not blinded to the procedure due to the impossibility of blinding two completely different impression methods. On the other hand, the participants were not blinded to the procedure (conventional or digital), but they were blinded to the intraoral scanner model (patients do not differentiate between iTero^TM^ and Primescan^TM^). This is an important limitation, which may have favored the results of the digital procedure over the conventional one, as it is more novel and technological and, therefore, more attractive to children and teenagers.

The main strength of our study is that it included a large group of children who had not previously experienced dental impressions; therefore, their comfort perception is genuine because, despite not being blinded for the study, it was their first contact with dental impressions, and both procedures were novel for the participants.

Dentists and other healthcare professionals must consider that digitalization and innovative technologies, such as artificial intelligence, are here to stay in the profession. Although the first investment for an intraoral scanner is high [9], since the cost calculation showed that the digital procedure is initially 10.7 times more expensive than the conventional one, the cost was balanced after 3.6 years of use with the conventional method. In a study carried out with university dental students in Germany, a preference was observed by students for the digital technique (3M True Definition), which indicates the importance of introducing digitalization in the impression procedure in university teaching [24]. In another study, students’ positive perspective on the implementation of digital dentistry in the preclinical curriculum is proven, although they prefer that the activities be evaluated by a teacher and not by artificial intelligence methods. Currently, more than 90% of students report imagining themselves using intraoral scanners to treat patients in their consultations [25]. Research groups should currently continue investigating methods to help with digitalization and reduce costs to be able to generalize their use.

## 5. Conclusions

Digital impressions are superior in terms of total comfort to conventional alginate impressions in children and adolescents. The perception of breathing, nausea, and gag reflex is significantly worse with conventional printing than with the scanners studied (iTero^TM^ and Primescan^TM^). Children and adolescents perceive the impression with iTero^TM^ as more painful than with Primescan^TM^.

## Figures and Tables

**Table 1:** Technical specifications of the electronic digital caliper according to manufacturer.

Measure range	0–75 mm/0–3″
	0–100 mm/0–4″
	0–150 mm/0–6″
	0–200 mm/0–8″
	0–300 mm/0–12″
Resolution	0.01 mm/0.0005″
Accuracy	±0.02 mm/0.001″ (<100 mm)
	±0.03 mm/0.001″ (>100–200 mm)
	±0.04 mm/0.0015″ (>200–300 mm)
Repeatability	0.01 mm/0.0005″
Measuring system	Linear capacitive measuring system
Working temperature	5~40 °C/41–104 °C
Influence of humidity	Not important under 80% of relative humidity

**Table 2:** Descriptive statistics and normality tests of the VAS scales of conventional impression comfort, iTero^TM^, and Primescan^TM^.

	VAS	Mean	95% CI^+^ for the Mean		SD ^#^	Test for Normal Distribution Sig. (*p*)	
	Item		Lower Limit	Upper Limit		Kolmogorov–Smirnov ^a^	Shapiro–Wilk
Conventional impression	1—General perception	15.75	9.58	21.93	21.95	<0.001 *	<0.001 *
	2—Breathing	18.58	11.56	25.61	24.96	<0.001 *	<0.001 *
	3—Smell–taste	19.79	12.13	27.64	27.56	<0.001 *	<0.001 *
	4—Hot–cold	13.73	8.81	19.66	17.51	<0.001 *	<0.001 *
	5—Nausea	23.30	14.79	31.81	30.25	<0.001 *	<0.001 *
	6—Gag reflex	23.89	15.79	31.98	28.77	<0.001 *	<0.001 *
	7—Pain	13.55	7.28	19.83	22.32	<0.001 *	<0.001 *
	Total comfort	18.37	12.97	23.78	19.21	0.001 *	<0.001 *
iTero^TM^	1—General perception	9.63	5.19	14.06	15.76	<0.001 *	<0.001 *
	2—Breathing	4.72	2.25	7.18	8.77	<0.001 *	<0.001 *
	3—Smell–taste	11.33	6.13	16.53	18.48	<0.001 *	<0.001 *
	4—Hot–cold	8.63	4.19	13.06	15.75	<0.001 *	<0.001 *
	5—Nausea	7.26	2.63	11.88	16.44	<0.001 *	<0.001 *
	6—Gag reflex	8.19	2.79	13.59	19.19	<0.001 *	<0.001 *
	7—Pain	11.57	6.37	16.77	18.49	<0.001 *	<0.001 *
	Total comfort	8.76	5.55	11.97	11.41	<0.001 *	<0.001 *
Primescan^TM^	1—General perception	1.93	0.45	3.41	5.25	<0.001 *	<0.001 *
	2—Breathing	1.67	−0.09	3.42	6.25	<0.001 *	<0.001 *
	3—Smell–taste	6.84	3.53	10.14	11.75	<0.001 *	<0.001 *
	4—Hot–cold	5.88	3.11	8.64	9.84	<0.001 *	<0.001 *
	5—Nausea	0.98	−0.23	2.20	4.33	<0.001 *	<0.001 *
	6—Gag reflex	0.49	−0.02	1.01	1.85	<0.001 *	<0.001 *
	7—Pain	2.73	0.96	4.49	6.29	<0.001 *	<0.001 *
	Total comfort	2.93	1.81	4.06	4.00	<0.001 *	<0.001 *

CI^+^, confidence interval. SD ^#^, standard deviation. ^a^ Lilliefors significance correction. * Statistically significant, *p* ≤ 0.05.

**Table 3:** Post-hoc paired comparison of VAS items for conventional impression, iTero^TM^, and Primescan^TM^.

VAS	Conventional vs. Primescan^TM^	Conventional vs. iTero^TM^	iTero^TM^ vs. Primescan^TM^
	Sig. (*p*) ^a^	Sig. (*p*) ^a^	Sig. (*p*) ^a^
1—General perception	0.003 *	0.704	0.113
2—Breathing	0.001 *	0.012 *	1
3—Smell–Taste	0.046 *	0.453	0.966
4—Hot–Cold	0.113	0.078	1
5—Nausea	<0.001 *	0.017 *	0.765
6—Gag reflex	<0.001 *	0.005 *	0.647
7—Pain	0.035 *	1	0.005 *
Total comfort	<0.001 *	0.001 *	0.040 *

^a^ Post-hoc tests for pairwise comparison between variables adjusted with the Bonferroni correction. * Statistically significant, *p* ≤ 0.05.

## Data Availability

The raw data presented in this study are available on request from the corresponding author (M.M.P.-C.) due to privacy restrictions. In addition, due to copyright requirements, the VAS scale translated into Spanish and used in the study is also available on request via email.
